# Malignant peritoneal mesothelioma presenting as a mass in a Spigelian hernia. Report of a case

**DOI:** 10.1016/j.ijscr.2020.03.005

**Published:** 2020-03-07

**Authors:** Paul H. Sugarbaker

**Affiliations:** Program in Peritoneal Surface Malignancies, MedStar Washington Hospital Center, 106 Irving St., NW, Suite 3900, Washington, DC, USA

**Keywords:** Tumor cell entrapment, Peritoneal metastases, HIPEC, Intraperitoneal chemotherapy, Cytoreductive surgery, Peritonectomy

## Abstract

**Background:**

Gastrointestinal cancer, gynecologic cancer and malignant peritoneal mesothelioma may disseminate widely to the surfaces of the abdomen and pelvis. In some patients this spread of the disease may find its way into a hernia sac.

**Methods:**

The Spigelian hernia is caused by a protrusion of the peritoneum of the anterior abdominal wall into the abdominal wall muscle. This peritoneal defect occurs through the semilunar line of Spiegel in or closely associated with the semicircular line of Douglas.

**Results:**

A patient presented with a large (11 cm greatest diameter) mass in the left lower quadrant. The mass was composed of malignant peritoneal mesothelioma and was anatomically located within the Spigelian hernia belt. At the time of cytoreductive surgery numerous other sites of malignant peritoneal mesothelioma were present within the abdomen and pelvis.

**Conclusions:**

The left lower quadrant mass was a malignant peritoneal mesothelioma presenting within a Spigelian hernia defect. This phenomenon has not been previously reported.

## Highlights

• Cancers that occur within the abdomen and pelvis may present within a hernia.
• Cancer presenting within a Spigelian hernia has not been previously reported.
• Malignant peritoneal mesothelioma presented within a Spigelian hernia.
• Peritoneal mesothelioma progressed rapidly within a hernia sac.

## 1 Introduction

When cancer disseminates within the abdominal and pelvic space as peritoneal metastases, it does so by a definite pattern that is characteristic of a particular cancer diagnosis [1]. Hernia defects of the parietal peritoneal lining of the abdomen and pelvis are sites for cancer cells to be trapped and then progress. In many different gastrointestinal and gynecologic malignancies, a tumor mass and/or malignant ascites causing a new onset hernia may be the presenting symptom or sign of the cancer [2].

Many different types of hernia defects of the parietal peritoneum exist and result in a protrusion of the skin of the abdominal wall. Over a lifetime, the prevalence of inguinal hernias is approximately 7.3% of men. The prevalence in women is approximately 5% of that in men [3]. Umbilical hernias occur at approximately one-fourth the prevalence of groin hernias and are twice as common in men as compared to women [3]. A more unusual abdominal wall hernia defect is the Spigelian hernia which occurs between 0.6% and 2% of all hernias of the abdominal wall [4,5].

A rare malignancy occurring as a result of cancerous change of the lining of the abdomen and pelvis is malignant peritoneal mesothelioma. It occurs in the United States in approximately 800 patients per year [6]. A new onset hernia has been reported to present as a first symptom/sign of malignant peritoneal mesothelioma in 13% of patients [7].

To my knowledge, the presenting sign of malignant peritoneal mesothelioma in a Spigelian hernia has not been previously reported. In this case report a 70-year-old woman presented with an abdominal wall mass of malignant peritoneal mesothelioma at the exact site where a Spigelian hernia would case a bulge. This is the first report of malignant peritoneal mesothelioma in a Spigelian hernia leading to the diagnosis and definitive treatment of this disease.

Data on this patient was prospectively recorded and then retrospectively reviewed at academic institutions. This research work has been reported in line with the SCARE criteria [8]. This study was registered as a case report on the www.researchregistry.com website with UIN 5346.

## 2 Case report

In November 2002, a 70-year-old white woman complained of pain in a left lower quadrant abdominal mass. By physical examination a mass was fixed within the abdominal wall musculature within the left lower quadrant of the abdomen.

**Fig. 1:**
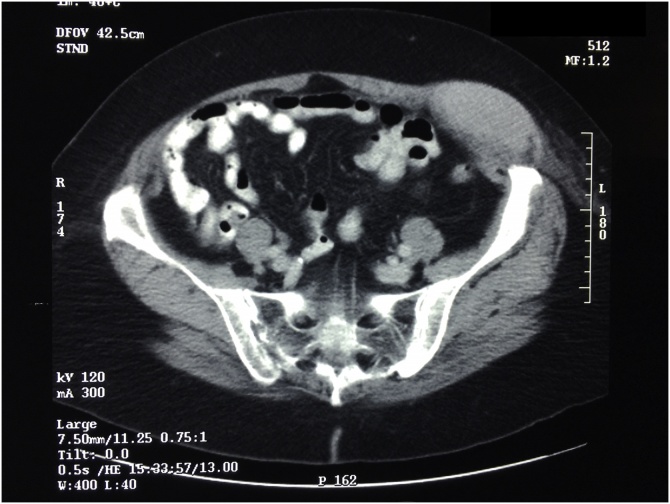
CT cut through the lower abdomen in a 70-year-old woman. Percutaneous biopsy revealed malignant mesothelioma. The tumor mass occurred within the Spigelian Hernia Belt and appeared to be expanding laterally.

On November 11, 2003, a CT was obtained which showed a mass within the anterior abdominal wall measuring 11 cm in diameter (Fig. 1). A second 5 cm by 2.9 cm mass was associated with the right colon. No disease was seen outside the abdomen and pelvis. Biopsy of the abdominal wall mass showed malignant peritoneal mesothelioma.

On March 4, 2003, a 9-h surgical procedure was performed which removed all visible evidence of mesothelioma from abdominal wall, abdomen and pelvis. Resection of the mass from the left lower aspect of the abdominal wall required resection of the lower one-half of the rectus abdominus muscle and adjacent abdominal wall musculature to the iliac crest. The resected specimen measured 15 cm × 12 cm × 7 cm.

Other sites of disease that needed to be removed in order to have a complete cytoreduction included a greater omentectomy and splenectomy, peritoneum from beneath the right hemidiaphragm, tumor nodules from small bowel and its mesentery, and complete pelvic peritonectomy with resection of rectosigmoid colon, uterus, Fallopian tubes and ovaries [9].

After complete cytoreduction the patient was treated with hyperthermic intraperitoneal chemotherapy (HIPEC) [10].

The patient’s postoperative course was uneventful. At 18 months postoperatively multiple lung metastases were seen by CT. The patient died of progressive disease in July of 2005, 29 months after her treatment by cytoreductive surgery and HIPEC.

## 3 Discussion

### 3.1 Origin of the abdominal wall tumor mass

A definitive determination of the pathophysiology of a malignant mesothelioma mass within the anterior abdominal wall in a 70-year-old woman is not obvious. Malignant peritoneal mesothelioma within the musculature of the abdominal wall does not occur unless there is a laparoscopy trochar site or a transabdominal biopsy of the malignant mesothelioma tumor [7]. It has been reported within hernia sites [7,11]. One may safely assume that malignant peritoneal mesothelioma may manifest itself within a Spigelian hernia. The unusual location of this malignant mesothelioma tumor in the left lower quadrant of the abdominal wall is at the anatomic site of a Spigelian hernia. This is the anatomic site where the semicircular line of Douglas intersects the semilunar line of Spiegel. Ninety percent of all Spigelian hernias occur within a few centimeters of this intersection, the anatomic site of the Spigelian hernia belt [12]. The lateralization of the mesothelioma mass as it expands is caused by a spreading of the soft muscle fibers of the internal oblique muscle [12]. Other explanations for the anatomic location of the large malignant mesothelioma tumor mass are not obvious.

### 3.2 Tumor progression in a hernia sac

There are several reports in the oncology literature regarding peritoneal metastases becoming symptomatic within a hernia sac. An early example of cancer cells and/or malignant ascites accumulating as a mass at the umbilical hernia defect was reported by Mayo et al. [13]. The umbilical mass was caused by endometrial cancer expansion within a small umbilical hernia defect. This has been referred to as the Sister Mary Joseph’s sign [14]. In patients with mucinous appendiceal neoplasms, 25% of males and 4% of females have a new onset abdominal wall hernia as the presenting sign. Two-thirds of these hernias are inguinal and one-third were umbilical. Spigelian hernias were not mentioned in this report [15]. In patients with malignant peritoneal mesothelioma, 17% of men and 5% of women were diagnosed because of a new onset inguinal hernia [7]. From these clinical data the occurrence of malignant peritoneal mesothelioma presenting as a painful mass in a Spigelian hernia is completely possible.

### 3.3 Distribution of malignant peritoneal mesothelioma into a hernia sac

In our patient, malignant peritoneal mesothelioma was widely distributed on the peritoneal surfaces of the abdomen and pelvis. By far, the largest tumor accumulation was within the Spigelian hernia sac. Tumor was present within the omentum in large volume likely because of phagocytic activity of omental macrophages. At other sites the malignant mesothelioma was layered out on the peritoneal surface of the right hemidiaphragm, surface of the spleen and pelvic peritoneal surfaces. The only large mass (15 cm × 12 cm × 7 cm) was within the hernia defect. Not surprising this painful mass in the left lower quadrant of the abdomen was this patient’s presenting complaint. Recently, we reported on a male malignant peritoneal mesothelioma patient whose presenting complaint was a left inguinal mass [11]. This symptomatic mass was several times larger than any other tumor present and was confirmed to be a solid mass of malignant peritoneal mesothelioma. The cause for a large tumor mass to be present within a hernia sac is not immediately apparent. It is possible that growth factors released by the tumor causes a paracrine type of stimulation of mitotic activity restricted to the confines of the hernia sac. Other explanations may be relevant to this observation regarding augmented tumor growth within a hernia sac.

## Declaration of Competing Interest

Paul H. Sugarbaker has no conflicts of interest to declare.

## Sources of funding

Data management and secretarial support provided by Foundation for Applied Research in Gastrointestinal Oncology.

## Ethical approval

Local IRB-approval for this case report was not required:

MedStar Health Institutional Review Board has determined that a case report of less than three [3] patients does not meet the DHHS definition of research (45 CFR 46.102(d)(pre-2018)/45 CFR 46.102(l)(1/19/2017)) or the FDA definition of clinical investigation (21 CFR 46.102(c)) and therefore are not subject to IRB review requirements and do not require IRB approval.

This case report is of 1 patient.

## Consent

Informed consent was requested through surviving family members for publication of this case report and accompanying images.

## Author’s contribution

Paul H. Sugarbaker, MD: study concept or design, data collection, data analysis or interpretation, writing the paper.

## Registration of research

This study was registered as a case report on the www.researchregistry.com website with UIN 5346.

## Guarantor

Paul H. Sugarbaker, MD

## Provenance and peer review

Not commissioned, externally peer-reviewed.

## References

[1] Carmignani P., Sugarbaker T.A., Bromley C.M., Sugarbaker P.H. (2003). Intraperitoneal cancer dissemination: mechanisms of the patterns of spread. Cancer Metastasis Rev..

[2] Sugarbaker P.H. (2017). Management of an inguinal hernia in patients with pseudomyxoma peritonei. Eur. J. Surg. Oncol..

[3] Rutkow I.M. (1998). Epidemiologic, economic, and sociologic aspects of hernia surgery in the United States in the 1990s. Surg. Clin. N. Am..

[4] Houlihan T.J. (1976). A review of Spigelian hernias. Am. J. Surg..

[5] Skandalakis P.N., Zoras O., Skandalakis J.E., Mirilas P. (2006). Spigelian hernia: surgical anatomy, embryology, and techniques of repair. Am. Surg..

[6] Sugarbaker P.H., Turaga K.K., Alexander H.R., Deraco M., Hesdorffer M. (2016). Management of malignant peritoneal mesothelioma using cytoreductive surgery and perioperative chemotherapy. J. Oncol. Pract..

[7] Sugarbaker P.H., Welch L., Mohamed F., Glehen O. (2003). A review of peritoneal mesothelioma at the Washington Cancer Institute. Surg. Oncol. Clin. N. Am..

[8] Agha R.A., Borrelli M.R., Farwana R., Koshy K., Fowler A., Orgill D.P., For the SCARE Group (2018). The SCARE 2018 statement: updating consensus Surgical CAse REport (SCARE) Guidelines. Int. J. Surg..

[9] Sugarbaker P.H., van der Speeten K., Sugarbaker P.H. (2017). An overview of peritonectomy, visceral resection, and therapeutic laparoscopy for peritoneal surface malignancy. Cytoreductive Surgery & Perioperative Chemotherapy for Peritoneal Surface Malignancy. Textbook and Video Atlas.

[10] Van der Speeten K., Stuart O.A., Sugarbaker P.H., Sugarbaker P.H. (2017). Cancer chemotherapy for peritoneal metastases: pharmacology and treatment. Cytoreductive Surgery & Perioperative Chemotherapy for Peritoneal Surface Malignancy. Textbook and Video Atlas.

[11] P.H. Sugarbaker, J.S. Jelinek, Unusual radiologic presentations of malignant peritoneal mesothelioma, Nova Science Publishers in press. 10.4329/wjr.v12.i12.316 PMC7802078 33510855

[12] Reardon P.R. (2012). Laparoscopic preperitoneal repair of Spigelian hernia utilizing mesh. ACS Multimedia Atlas of Surgery: Hernia Surgery Volume.

[13] Bailey H. (1949). Demonstrations of Physical Signs in Clinical Surgery.

[14] Sugarbaker P.H. (2001). Sister Mary Joseph’s sign from metastatic disease of the pancreas. J. Am. Coll. Surg..

[15] Esquivel J., Sugarbaker P.H. (2000). Clinical presentation of the pseudomyxoma peritonei syndrome. Br. J. Surg..

